# Safety of immune checkpoint inhibitors in a diverse patient population: a single-institution experience

**DOI:** 10.3332/ecancer.2026.2150

**Published:** 2026-06-18

**Authors:** Grace M Ferri, John F Murphy, Akash Oza, Alexander J B Bulteel, Wafaa Abbasi, Rachel Anderson, Mehmed Taha Dinc, Eva Gaufberg, Kayra Cengiz, Sainikhil Sontha, Janice Weinberg, Patrick Kurpaska, Yashvin Onkarappa Mangala, Matthew Kulke, Umit Tapan

**Affiliations:** 1 Section of General Internal Medicine, Department of Medicine, Boston Medical Center, Boston, MA 02215, USA; 2 Boston University Chobanian and Avedisian School of Medicine, Boston, MA 02215, USA; 3 Department of Biostatistics, Boston University School of Public Health, Boston, MA 02215, USA; 4 Section of Hematology and Oncology, Boston University Chobanian and Avedisian School of Medicine and Boston Medical Center, Boston, MA 02215, USA

**Keywords:** immunotherapy, antineoplastic agents, drug-induced abnormalities, medical oncology, diversity, equity, inclusion

## Abstract

**Introduction:**

Among racial and socioeconomic minorities not appropriately represented by trial populations, rates of immune-related adverse events (irAEs) are unclear.

**Objective:**

We sought to characterise factors associated with irAEs in adult cancer patients at Boston Medical Center, a safety-net hospital serving a diverse patient population, including racial and ethnic minorities.

**Methods:**

We performed a retrospective study on all adult cancer patients at Boston Medical Center treated with first-line immune checkpoint inhibitors (ICIs) between 7/1/15 and 6/30/22. Baseline demographic (including socioeconomic) and oncologic variables were collected.

**Results:**

From the overall cohort (n = 469), 34 patients (n = 34, 7%) on first-line ICI without prior chemotherapy developed at least one ICI toxicity. Toxicity was experienced in 10% of White patients, 5% of Black patients, 7% of Hispanic patients, 7% of non-Hispanic patients, 9% of public insurance recipients and 7% of private insurance recipients. When comparing those who sustained irAEs (n = 34) relative to their counterparts (n = 435), Fisher’s exact test did not indicate any significant association between incidence of irAEs and gender (p = 0.853), race (p = 0.352), ethnicity (p = 1.00), language (p = 0.827), insurance (p = 1.00), education (p = 0.267) or smoking (p = 0.695).

**Conclusion:**

We did not identify disproportionate rates of toxicity among patients of racial and ethnic minorities or with public insurance. These findings suggest that management decisions and toxicity risk assessment should not be based solely on race or socioeconomic status. Future studies featuring diverse patient populations must be conducted to formulate clinical algorithms for irAE management.

## Introduction

The therapeutic potential of immune checkpoint inhibitors (ICIs) in treating malignancies and improving survival has been established in clinical trials [1, 2]. However, cited studies demonstrate low rates of enrollment of minority populations within oncology drug clinical trials [3–13]. As a result, we lack sufficient data on ICI efficacy and safety within racial and socioeconomic minorities under-represented by trial populations [7, 14]. While studies have attempted to explore these side effect profiles within minority patient populations, data remain limited and contradictory [7, 15–17].

Herein we assessed patients with solid malignancies at a single institution who were (a) treated with first-line ICI without prior chemotherapy and (b) subsequently developed immune-related adverse events (irAEs) to gauge the impact of ethnicity and socioeconomic status on clinical outcomes. The study institution, Boston Medical Center Healthcare System, is the largest safety-net hospital in New England. Of the patients at Boston Medical Center, approximately 72% would be categorised as underserved. Patients belong to racial and ethnic minorities, where 32.1% of patients identify as Black, 5.2% as Asian, 0.3% as American Indian or Alaska Native, 15.2% as Hispanic or Latino and 0.2% as Native Hawaiian/Pacific Islander [18]. In addition, patients are often elderly, low-income or non-English-speaking (32%) and dependent on Medicaid or Medicare for health insurance coverage [19].

## Methods

We queried the electronic medical record through the Clinical Data Warehouse to retrospectively assess all patients aged 18 and above at Boston Medical Center with solid malignancies treated with ICIs between 1 July 2015 and 30 June 2022. From these initial 469 patients meeting inclusion criteria, we identified 34 patients who (a) were receiving first-line mono- or dual-ICI therapy, (b) had not received prior chemotherapy and/or ICIs and (c) had developed one or greater irAE(s). ICI toxicities (irAEs) were determined and graded through a detailed retrospective chart review based on the treating clinician’s assessment and documentation. In cases where the grade of the irAE was not documented, grading was determined by the physician conducting the chart review based on ASCO guidelines incorporating laboratory abnormalities and radiographic findings [20]. When grading was ambiguous, events were adjudicated by dual physician review to ensure accuracy. This study was deemed exempt by the Institutional Review Board of Boston Medical Center.

Baseline demographic data and oncologic data were collected on all 469 patients. Outcomes included the number of toxicities per patient, days to toxicity onset, grade of ICIs toxicity, treatment of ICI toxicity, length of treatment of ICI toxicity and outcome of ICI toxicity (continuation, interruption or discontinuation of ICI). Analyses including confidence intervals at the 95% threshold and Fisher’s exact test were performed in R.

## Results

From 1 July 2015 to 30 June 2022, 34 adult cancer patients at Boston Medical Center on first-line ICI without prior chemotherapy exposure developed at least one ICI toxicity. Demographic variables are outlined in Table 1 for these patients (*n* = 34) relative to the overall cohort of patients treated with ICIs (*n* = 469). As detailed in Supplementary Table 1, the most common underlying malignancies included lung cancer, melanoma, renal cell carcinoma, hepatocellular carcinoma and head and neck cancers. ICIs used included PD-1/PD-L1 inhibitors (e.g., nivolumab, pembrolizumab and cemiplimab) as well as CTLA-4 inhibition (ipilimumab), including combination therapy. Major organ irAEs included colitis, hepatitis, pneumonitis and nephritis, most arising within 250 days of treatment and meeting criteria for grade 2–3 toxicity. A substantial proportion required corticosteroids and higher-grade toxicities frequently led to interruption or discontinuation of ICI therapy. One fatality was attributed to ICI pneumonitis.

Toxicity was experienced in 7% of male patients (95% confidence interval (CI): 4%–11%) and 8% of female patients (95% CI: 4%–13%). In terms of race and ethnicity, toxicity was experienced in 10% of White patients (95% CI: 6%–15%), 5% of Black patients (95% CI: 3%–9%), 7% of Hispanic patients (95% CI: 3%–16%) and 7% of non-Hispanic patients (95% CI: 5%–10%). By language, toxicity was experienced in 7% of English-speaking patients (95% CI: 5%–11%), 5% of Spanish-speaking patients (95% CI: 1%–17%) and 6% of Haitian Creole-speaking patients (95% CI: 1%–27%). Toxicity was noted in 6% of current smokers, 8% of former smokers and 7% of never smokers. In terms of insurance, toxicity was documented in 9% of public insurance recipients and 7% of private insurance recipients. Toxicities were found in 0% of patients who had no schooling, 4% of patients who had completed 8th grade, 6% of patients who had completed some high school, 11% of patients who had graduated high school or passed the General Educational Development (GED) test, 7% of people who had completed some college and 6% of those who had graduated college.

When comparing those who did sustain irAEs (*n* = 34) relative to their counterparts (*n* = 435), results of the Fisher’s exact test did not indicate a significant association between incidence of irAEs and gender (*p* = 0.853), race (*p* = 0.352), ethnicity (*p* = 1.00), language (*p* = 0.827), insurance (*p* = 1.00), education (*p* = 0.267) or smoking (*p* = 0.695).

## Discussion

Our study did not identify disproportionate rates of toxicity among patients of racial and ethnic minorities or with public insurance. To our knowledge, this study is the first to identify and study a novel cohort of socioeconomically diverse patients with irAEs on first-line ICIs without prior chemotherapy. This work builds on prior studies assessing racial and ethnic differences in response to PD-1/PD-L1 inhibitors in non-small cell lung cancer (NSCLC) and impact of insurance on treatment outcomes in patients on ICI therapy [15, 25].

Previous studies have analyzed patient health data prior to ICI therapy and created predictive toxicity-effectiveness modeling [21]. These models set the stage for clinical decision-making algorithms surrounding ICI use, monitoring and precautions. However, no studies have compared the frequency of irAEs in a population not exposed to chemotherapy, which can also cause multi-organ toxicities on unpredictable timescales [22, 23]. By eliminati﻿ng potential alternative sources of confounding bias (e.g., prior chemotherapy or chemoimmunotherapy), we sought to more appropriately correlate irAEs with predisposing risk factors.

This study is limited by a small sample size and power owing to these stringent exclusion criteria. Moreover, we could not appropriately characterise ICI efficacy or correlate irAEs with outcome, given the variety of cancers studied, the observational nature of this study and the small sample size. The observed rate of irAEs in our cohort (7%) is lower than rates reported in clinical trials (approximately 15%–30%) [24]. This difference likely reflects the retrospective design of our study, which may underestimate lower-grade toxicities that are under-documented in routine clinical practice, as well as differences in cohort selection, as we included only patients receiving first-line ICI without prior chemotherapy. Additionally, clinical trials employ structured toxicity monitoring, which may increase detection of lower-grade events.

Despite these limitations, our findings provide real-world insight into irAE incidence in a socioeconomically diverse population underrepresented in clinical trials. Prior small studies on minority populations with metastatic NSCLC do demonstrate decreased cumulative dose of ICI and decreased survival [25, 26]. These adverse outcomes may be attributable to differences in management practices rather than intrinsic differences in medication efficacy or toxicity. Accordingly, we advocate for equitable ICI management regardless of race or socioeconomic status. We encourage larger studies on the incidence of irAEs among patients treated exclusively with ICI to create a more precise estimation of which toxicities are attributable to the ICI themselves. Future studies should include system-specific comorbidities predisposing to toxicity (e.g., history of dermatologic conditions among those with ICI dermatitis). Most importantly, diverse populations, such as the cohort we feature in this study, must be highlighted to promote in socially conscious care strategies.

## Conflicts of interest

The authors declare no potential conflicts of interest. No disclaimers or relevant financial disclosures.

## Funding

This work, including the preparation of this manuscript, was supported by Boston University Interdisciplinary Affinity Research Collaborative funding awarded to Dr. Matthew Kulke.

## Author contributions

Conceptualisation, investigation, methodology, project administration, supervision - GMF, JFM, AO, AJBB, UT, YM, PK; data curation - KC, WA, EG, RA, MD, GMF, JFM, AO; formal analysis - JW, GMF; writing - GMF, JFM, AO, SS; funding acquisition - MK.

## Data availability

The data underlying this article will be shared on reasonable request to the corresponding author.

## Figures and Tables

**Supplementary Table 1.** Outcomes by major organ toxicity among patients with irAEs (n = 34).

	Nephritis (*n* = 2)	Colitis (*n* = 9)	Hepatitis (*n* = 5)	Pneumonitis (*n* = 5)
Cancer type, *n* (%)				
Cervical	0 (0%)	0 (0%)	0 (0%)	0 (0%)
Cutaneous squamous cell carcinoma	0 (0%)	0 (0%)	1 (20%)	0 (0%)
Head and neck cancer	0 (0%)	0 (0%)	1 (20%)	1 (20%)
Hepatocellular carcinoma	0 (0%)	1 (11%)	1 (20%)	1 (20%)
Lung	1 (50%)	2 (22%)	0 (0%)	2 (40%)
Melanoma	1 (50%)	5 (56%)	1 (20%)	1 (20%)
Merkel cell carcinoma	0 (0%)	0 (0%)	0 (0%)	0 (0%)
Renal cell carcinoma	0 (0%)	1 (11%)	1 (20%)	0 (0%)
Days to toxicity onset, Mean ± SD	56 ± 49	241 ± 435	91 ± 48	245 ± 254
Grade (%)				
1	1 (50%)	0 (0%)	1 (20%)	0 (0%)
2	0 (0%)	5 (56%)	0 (0%)	2 (40%)
3	1 (50%)	4 (44%)	4 (80%)	1 (20%)
4	0 (0%)	0 (0%)	0 (0%)	1 (20%)
5	0 (0%)	0 (0%)	0 (0%)	1 (20%)
ICI, *n* (%)				
Nivolumab	0 (0%)	2 (22%)	0 (0%)	1 (20%)
Pembrolizumab	2 (100%)	2 (22%)	1 (20%)	1 (20%)
Cemiplimab	0 (0%)	0 (0%)	1 (20%)	0 (0%)
Ipilimumab	0 (0%)	2 (22%)	1 (20%)	0 (0%)
Ipilimumab/Nivolumab	0 (0%)	3 (33%)	2 (40%)	0 (0%)
ICI outcome, *n* (%)				
Continuation	0 (0%)	1 (11%)	1 (20%)	0 (0%)
Interruption	0 (0%)	1 (11%)	0 (0%)	0 (0%)
Discontinuation	2 (100%)	7 (78%)	4 (80%)	5 (100%)

Abbreviations: irAEs = immune-related adverse events; SD = standard deviation; ICI = immune checkpoint inhibitor

**Table 1.** Baseline characteristics by irAE status (*n* = 469).

	All patients receiving ICI(s) (n = 469)	Patients with irAE(s) on first-line ICI(s) (n = 34)
Gender, *n* (%)		
Male	309 (66%)	22 (65%)
Female	160 (34%)	12 (35%)
Race, *n* (%)*		
White	185 (39%)	18 (53%)
Black	196 (42%)	10 (29%)
Asian	32 (7%)	2 (6%)
Asian Indian	4 (1%)	0 (0%)
American Indian/Native American	3 (1%)	0 (0%)
Ethnicity, *n* (%)**		
Hispanic	59 (13%)	4 (12%)
Not Hispanic	409 (87%)	30 (88%)
Language, *n* (%)		
English	341 (73%)	25 (74%)
Spanish	37 (9%)	3 (9%)
Cape Verdean/Port Creole	12 (3%)	1 (3%)
Haitian Creole	17 (4%)	1 (3%)
Vietnamese	18 (4%)	2 (6%)
Nepali	5 (1%)	0 (0%)
Portuguese	5 (1%)	0 (0%)
Other#	34 (7.2%)	2 (6%)
Smoking status, *n* (%)**		
Current	102 (22%)	6 (18%)
Former	224 (48%)	18 (53%)
Never	139 (30%)	10 (29%)
Insurance***		
Public	339 (92%)	31 (94%)
Private	28 (8%)	2 (6%)
Highest grade completed, *n* (%)***		
No schooling	32 (7%)	0 (%)
8th grade	45 (10%)	2 (6%)
Some high school	89 (20%)	5 (16%)
Graduated high school/GED	188 (43%)	20 (63%)
Some college	30 (7%)	2 (6%)
Graduated college	51 (12%)	3 (9%)

*Data not available for 12% (4/34) patients on race, 3% (1/34) patients on insurance and 6% (2/34) patients on highest grade completed

**Data not available for 0.2% (1/469) patients on ethnicity, 1% (4/469) on smoking status, 22% (102/469) on insurance and 7% (34/469) on highest grade completed

^#^Other languages include Cambodian, French, Chinese/Cantonese, Chinese/Mandarin, Laotian, Amharic/Ethiopian, Somali, Hindi, Russian, Yoruba, Tagalog, Hungarian, Armenian, Polish, Arabic, Albanian

Abbreviations: ICI = immune checkpoint inhibitor, irAE = immune-related adverse event, GED = General Education Development (test)
